# De-escalating chemotherapy for stage I–II gastric neuroendocrine carcinoma? A real-world competing risk analysis

**DOI:** 10.1186/s12957-023-03029-2

**Published:** 2023-05-06

**Authors:** Danwei Du, Yangyang Xie, Xiaowen Li, Zhongkai Ni, Jinbo Shi, Hai Huang

**Affiliations:** Department of General Surgery, Hangzhou TCM Hospital Affiliated to Zhejiang Chinese Medical University, Zhejiang Province, Hangzhou, 310000 China

**Keywords:** SEER Program, Nomogram, Chemotherapy, Gastric cancer, Competing risk analyses

## Abstract

**Background:**

The role of adjuvant chemotherapy in gastric neuroendocrine neoplasms (GNEC) has not been well clarified yet. The study was designed to investigate the potential effect of adjuvant chemotherapy in stage I–II GNEC patients and construct a predictive nomogram.

**Method:**

Stage I–II GNEC patients were included in the Surveillance, Epidemiology, and End Results (SEER) database and divided into chemotherapy and no-chemotherapy groups. We used Kaplan–Meier survival analyses, propensity score matching (PSM), and competing risk analyses. The predictive nomogram was then built and validated.

**Results:**

Four hundred four patients with stage I–II GNEC were enrolled from the SEER database while 28 patients from Hangzhou TCM Hospital were identified as the external validation cohort. After PSM, similar 5-year cancer-specific survival was observed in two groups. The outcomes of competing risk analysis indicated a similar 5-year cumulative incidence of cancer-specific death (CSD) between the two cohorts (35.4% vs. 31.4%, *p* = 0.731). And there was no significant relation between chemotherapy and CSD in the multivariate competing risks regression analysis (HR, 0.79; 95% CI, 0.48–1.31; *p* = 0.36). Furthermore, based on the variables from the multivariate analysis, a competing event nomogram was created to assess the 1-, 3-, and 5-year risks of CSD.

The 1-, 3-, and 5-year area under the receiver operating characteristic curve (AUC) values were 0.770, 0.759, and 0.671 in the training cohort, 0.809, 0.782, and 0.735 in the internal validation cohort, 0.786, 0.856, and 0.770 in the external validation cohort.

Furthermore, calibration curves revealed that the expected and actual probabilities of CSD were relatively consistent.

**Conclusion:**

Stage I–II GNEC patients could not benefit from adjuvant chemotherapy after surgery. De-escalation of chemotherapy should be considered for stage I–II GNEC patients. The proposed nomogram exhibited excellent prediction ability.

**Supplementary Information:**

The online version contains supplementary material available at 10.1186/s12957-023-03029-2.

## Introduction

Gastric cancer (GC) is a highly heterogeneous malignancy, including various pathologies which present significantly different molecular patterns, tumor behavior, and prognoses [1]. Gastric neuroendocrine carcinoma (GNEC) is a rare histology type of GC, which occupies 0.1 to 0.6% of whole patients, with an increasing trend over the past few decades [2]. GNEC is a poorly differentiated, high-grade malignancy, which presents dismal prognoses and is prone to distant metastasis [3]. Compared with gastric adenocarcinoma, many studies have observed worse survival in GNEC patients [4, 5].

Based on the Japanese Classification of Gastric Carcinoma, a frequent treatment plan for GNEC is a combination of radical surgery and adjuvant chemotherapy [6]. Xie et al. retrospectively analyzed clinical data from a single center, indicating that stage I-III GNEC patients could benefit from adjuvant chemotherapy (median survival time 43 months vs. 13 months, *p* = 0.026) [7]. However, a Chinese study recruited 804 GNEC patients from 21 centers between 2004 and 2016, indicating that stage I–II patients could not obtain improved prognoses after adjuvant chemotherapy based on platinum or 5-fluorouracil [8]. There are still no clear clinical guidelines or consensuses that focus on this issue. With the popularity of gastroscopy and other early screening methods, the incidence of early-stage GNEC is increasing. Whether these patients can benefit from adjuvant chemotherapy remain controversial.

A nomogram, also known as a visual risk regression model, is a tool that predicts a specific clinical result, or the probability of a certain event based on the values of multiple clinical indicators and biological data. Despite the development of computer numerical calculation, nomograms demonstrate the advantages of convenience, simplicity, and practicality. They have been used widely to predict prognosis in various tumors, such as colon cancer, breast cancer, thyroid cancer, and so on [9–11]. However, to the best of our knowledge, a nomogram to predict the survival of patients with stage I–II GNEC patients has not been reported.

Based on the Surveillance, Epidemiology, and End Results (SEER) database, the retrospective research was designed to investigate the potential effect of adjuvant chemotherapy in stage I–II GNEC patients and construct a predictive nomogram.

## Materials and methods

### Data source and patient selection

Patients’ data were retrieved from the Surveillance, Epidemiology, and End Results (SEER) 18 Regs Custom Data Set (with additional treatment fields, November 2018 Sub) during 2010–2015. And the external validation data was extracted from the Hangzhou TCM Hospital from January 2012 to December 2016.

**Fig. 1:**
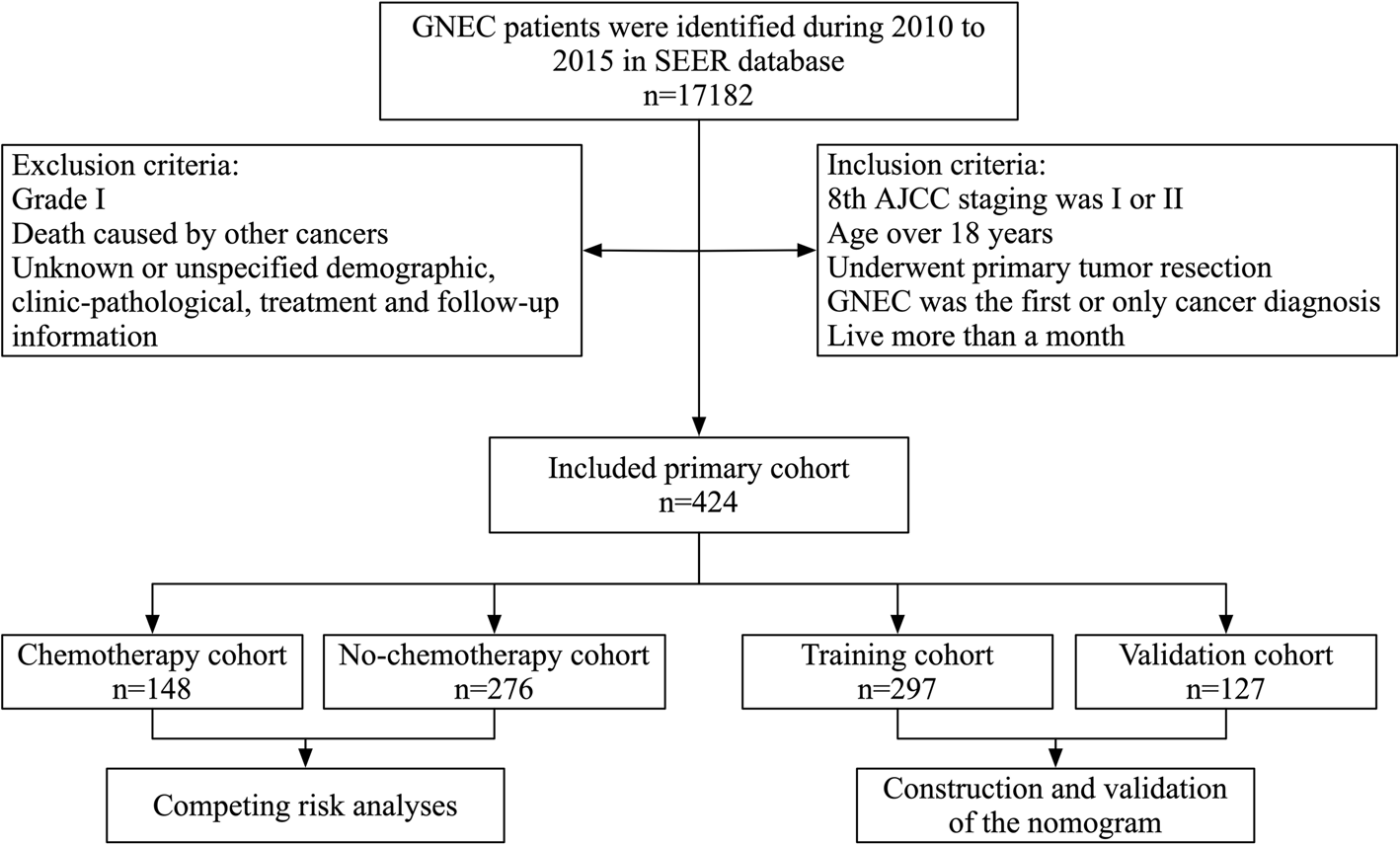
The detailed process of patient selection

The inclusion criteria were as follows: (1) age over 18 years; (2) GNEC was the first or only cancer diagnosis; (3) patients underwent surgical resection of tumor; (4) patients who lived more than a month; (5) patients with GNEC were identified using the third edition of the International Classification of Diseases for Oncology (ICD-O-3) (8012, 8013, 8041, 8042, 8043, 8044), including small cell neuroendocrine carcinoma (SCNEC) and large cell neuroendocrine carcinoma (LCNEC); (6) 8th AJCC staging was I or II. And the exclusion criteria were as follows: (1) death caused by other cancers; (2) patients with a history of other malignancies; (3) grade of I; (4) patients with incomplete demographic, clinicopathological, therapy, or follow-up data were eliminated from the research. Figure 1 presents the detailed procedure of patient selection.

### Clinicopathological variables

Clinical variables included year at diagnosis, age, gender, race, marital status, grade, AJCC stage, pathology, T stage, N stage, primary site, tumor size, regional nodes examined (RNE), surgery, chemotherapy, survival months, and survival status. Patients were categorized according to the primary site (cardia, distal site, middle site, and overlapping/NOS), tumor size (≤ 2 cm, 2–5 cm, and > 5 cm), and RNE (0, 1–15, and ≥ 16). The 7th edition of the AJCC TNM staging system was converted to the 8th edition based on the SEER database.

### Statistical analysis

The chi-square test was applied to compare categorical variables. Overall survival (OS) and cancer-specific survival (CSS) were compared using Kaplan–Meier survival analyses. Patients were split into three endpoints of interest to precisely examine the possible effect of competing risk factors: alive, cancer-specific death (CSD), and other causes death (OCD). The R package “cmprsk” was used to perform cumulative incidence function analyses and construct Fine and Grey’s proportional subdistribution hazard model [12].

The propensity score matching (PSM) approach was a novel statistical strategy that could reduce confounding factors in the study [13]. The match ratio of patients in both chemotherapy and no-chemotherapy groups was 1:1 using the nearest-neighbor algorithm within a caliper of 0.1. All the covariates used for matching in the research were as follows: year at diagnosis, age, gender, race, marital status, grade, AJCC stage, pathology, T stage, N stage, primary site, tumor size, and RNE. We utilized standardized difference (SD) to show how variables changed before and after PSM. SD ≤ 0.1 indicated ideal balances after match [14]. This method was carried out by the R package “matching”.

The patients were then randomly separated into two groups: training (70%) and validation (30%). The competing risk model's prognostic parameters were used to build a 1-, 3-, and 5-year CSD nomogram in the training dataset. The detailed procedure was based on Zhang’s research [15]. The area under the receiver operating characteristic curve (AUC) values and calibration plots were used to assess the predictive ability of the model. In the calibration curves, 1000 bootstrap resamples were utilized to compare the expected and observed survival probabilities. The receiver operating characteristic (ROC) curves were also displayed to illustrate the prediction capacity of the model and calculate AUC.

R software, version 4.0.3 (http://www.r-project.org), was used for all statistical analyses and visualization. A two-tailed *p* < 0.05 was determined to be statistically significant.

## Results

Four hundred four stage I-II GNEC patients were enrolled in the SEER database from 2010 to 2015, while 28 patients from Hangzhou TCM Hospital were identified as the external validation cohort. 148 of these patients received chemotherapy, whereas the remaining 276 had not. Age, gender, AJCC stage, grade, T stage, N stage, primary site, tumor size, and RNE were significantly different in the two cohorts (all *p* < 0.05). Patients in the chemotherapy group were more likely to be younger, and married, and had higher histologic grade, higher T stage, higher N stage, and a higher proportion of large tumor size.

PSM was then performed to reduce the discrepancy of baseline data between groups. Figure S1 showed that SD in most parameters was less than 0.1, which indicated good balancing performance. Finally, 186 patients were divided into two groups: chemotherapy (*n* = 93) and no-chemotherapy (*n* = 93). Table 1 shows the baseline characteristics before and after PSM.

**Table 1:** The descriptive characteristics of stage I–II GNEC patients before and after PSM

Characteristics	Before PSM			*P* value	After PSM			*P* value
	All	Chemo	None		All	Chemo	None	
	*N* = 424	*N* = 148	*N* = 276		*N* = 186	*N* = 93	*N* = 93	
Year at diagnosis:				0.637				1
2010–1012	164 (38.7%)	60 (40.5%)	104 (37.7%)		71 (38.2%)	36 (38.7%)	35 (37.6%)	
2013–2015	260 (61.3%)	88 (59.5%)	172 (62.3%)		115 (61.8%)	57 (61.3%)	58 (62.4%)	
Age:				0.01				0.493
≤ 60	92 (21.7%)	43 (29.1%)	49 (17.8%)		45 (24.2%)	25 (26.9%)	20 (21.5%)	
> 60	332 (78.3%)	105 (70.9%)	227 (82.2%)		141 (75.8%)	68 (73.1%)	73 (78.5%)	
Gender:				< 0.001				0.874
Female	166 (39.2%)	37 (25.0%)	129 (46.7%)		58 (31.2%)	30 (32.3%)	28 (30.1%)	
Male	258 (60.8%)	111 (75.0%)	147 (53.3%)		128 (68.8%)	63 (67.7%)	65 (69.9%)	
Race:				0.522				0.765
Non-White	188 (44.3%)	62 (41.9%)	126 (45.7%)		75 (40.3%)	36 (38.7%)	39 (41.9%)	
White	236 (55.7%)	86 (58.1%)	150 (54.3%)		111 (59.7%)	57 (61.3%)	54 (58.1%)	
Marital status:				0.054				1
Married	244 (57.5%)	95 (64.2%)	149 (54.0%)		115 (61.8%)	57 (61.3%)	58 (62.4%)	
Unmarried	180 (42.5%)	53 (35.8%)	127 (46.0%)		71 (38.2%)	36 (38.7%)	35 (37.6%)	
AJCC stage:				< 0.001				0.877
I	202 (47.6%)	37 (25.0%)	165 (59.8%)		64 (34.4%)	31 (33.3%)	33 (35.5%)	
II	222 (52.4%)	111 (75.0%)	111 (40.2%)		122 (65.6%)	62 (66.7%)	60 (64.5%)	
Grade:				0.011				0.557
II	257 (60.6%)	77 (52.0%)	180 (65.2%)		93 (50.0%)	49 (52.7%)	44 (47.3%)	
III/IV	167 (39.4%)	71 (48.0%)	96 (34.8%)		93 (50.0%)	44 (47.3%)	49 (52.7%)	
Pathology:				0.165				0.55
SCNEC	237 (55.9%)	90 (60.8%)	147 (53.3%)		111 (59.7%)	58 (62.4%)	53 (57.0%)	
LCNEC	187 (44.1%)	58 (39.2%)	129 (46.7%)		75 (40.3%)	35 (37.6%)	40 (43.0%)	
T stage:				< 0.001				0.902
T1	170 (40.1%)	33 (22.3%)	137 (49.6%)		52 (28.0%)	25 (26.9%)	27 (29.0%)	
T2	104 (24.5%)	39 (26.4%)	65 (23.6%)		45 (24.2%)	24 (25.8%)	21 (22.6%)	
T3	131 (30.9%)	67 (45.3%)	64 (23.2%)		75 (40.3%)	38 (40.9%)	37 (39.8%)	
T4	19 (4.5%)	9 (6.1%)	10 (3.6%)		14 (7.5%)	6 (6.5%)	8 (8.6%)	
N stage:				< 0.001				0.856
N0	293 (69.1%)	65 (43.9%)	228 (82.6%)		104 (55.9%)	52 (55.9%)	52 (55.9%)	
N1	108 (25.5%)	70 (47.3%)	38 (13.8%)		66 (35.5%)	32 (34.4%)	34 (36.6%)	
N2/N3	23 (5.4%)	13 (8.8%)	10 (3.6%)		16 (8.6%)	9 (9.7%)	7 (7.5%)	
Primary site:				0.007				0.825
Cardia	28 (6.6%)	18 (12.2%)	10 (3.6%)		16 (8.6%)	8 (8.6%)	8 (8.6%)	
Distal site	190 (44.8%)	58 (39.2%)	132 (47.8%)		78 (41.9%)	41 (44.1%)	37 (39.8%)	
Middle site	148 (34.9%)	52 (35.1%)	96 (34.8%)		70 (37.6%)	32 (34.4%)	38 (40.9%)	
Overlapping/NOS	58 (13.7%)	20 (13.5%)	38 (13.8%)		22 (11.8%)	12 (12.9%)	10 (10.8%)	
Tumor size:				< 0.001				0.506
≤ 2 cm	137 (32.3%)	35 (23.6%)	102 (37.0%)		51 (27.4%)	25 (26.9%)	26 (28.0%)	
≤ 5 cm	189 (44.6%)	60 (40.5%)	129 (46.7%)		80 (43.0%)	37 (39.8%)	43 (46.2%)	
> 5 cm	98 (23.1%)	53 (35.8%)	45 (16.3%)		55 (29.6%)	31 (33.3%)	24 (25.8%)	
RNE:				0.002				0.929
> 16	185 (43.6%)	79 (53.4%)	106 (38.4%)		92 (49.5%)	47 (50.5%)	45 (48.4%)	
0	40 (9.4%)	6 (4.1%)	34 (12.3%)		11 (5.9%)	5 (5.4%)	6 (6.5%)	
1–15	199 (46.9%)	63 (42.6%)	136 (49.3%)		83 (44.6%)	41 (44.1%)	42 (45.2%)	

### Survival analysis

**Fig. 2:**
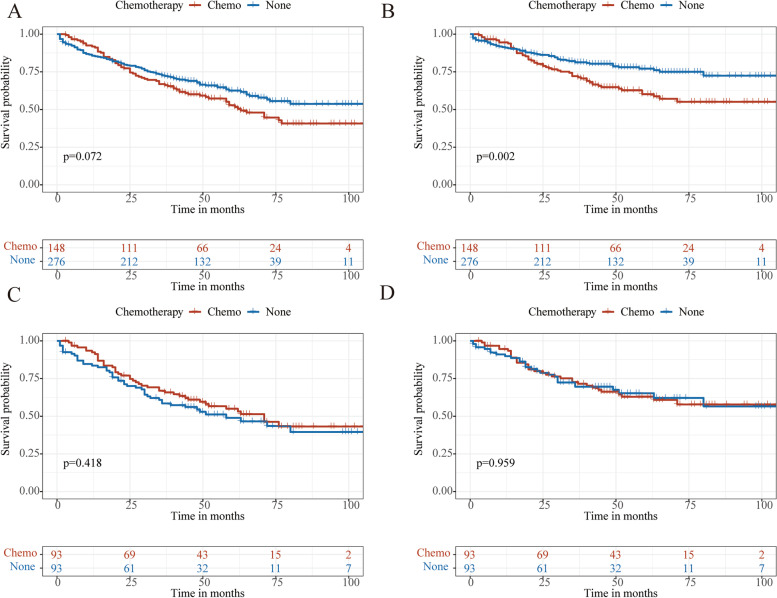
KM analyses of stage I-II GNEC patients. OS (**A**) and CSS (**B**) curves before PSM. OS (**C**) and CSS (**D**) curves after PSM

Before PSM, the patients without chemotherapy presented better CSS than the chemotherapy group, while no significant difference in OS was observed (Fig. 2A, B). After PSM, there was no significant discrepancy in OS and CSS between the two cohorts (Fig. 2C, D).

Considering the competing risk factors, cumulative incidence plots were further constructed. Compared with the no-chemotherapy cohort, GENC patients who underwent adjuvant chemotherapy suffered a higher 5-year cumulative incidence of CSD (37.8% vs. 21.3%, *p* = 0.001) and lower 5-year cumulative incidence of OCD rate (9.3% vs. 16.2%, *p* = 0.121) (Table 2). Subsequently, the subgroup analyses for AJCC stage, grade, tumor size, and pathology were conducted. The outcomes presented that the patients who received chemotherapy suffered significantly higher CSD in AJCC I stage, grade II, tumor size less than 2 cm, and SCNEC subgroups (all *p* < 0.05) (Figure S2). However, no equivalent results were observed when referring to other subgroups. Chemotherapy and CSD had no significant correlation in the multivariable competing risks regression analysis (HR, 0.79; 95% CI, 0.5–1.24; *p* = 0.3) (Table S1).

**Table 2:** The cumulative incidence of CSD and OCD in two cohorts before and after PSM

	Cancer-specific death (%)			*P* value	Other causes death (%)			*P* value
	1-year CIF	3-year CIF	5-year CIF		1-year CIF	3-year CIF	5-year CIF	
Before PSM								
None	0.088	0.178	0.213	0.001	0.055	0.096	0.162	0.121
Chemo	0.055	0.269	0.378		0.028	0.062	0.093	
After PSM								
None	0.099	0.281	0.314	0.731	0.066	0.134	0.197	0.133
Chemo	0.055	0.264	0.354		0.022	0.066	0.097	

*CIF* Cumulative incidences function

**Fig. 3:**
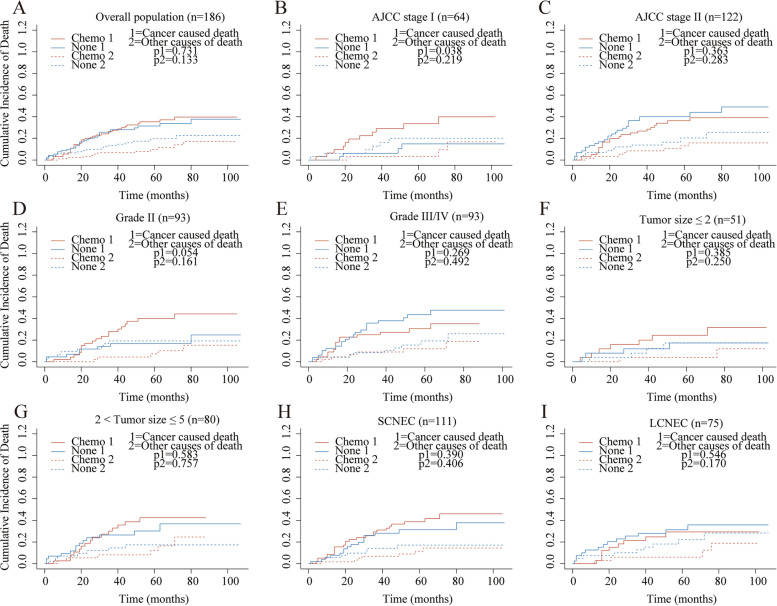
Cumulative incidence curves for the stage I–II GNEC patients in overall patients and subgroups after PSM. Overall patients (**A**), stage I (**B**), stage II (**C**), grade II (**D**), grade III/IV (**E**), tumor size ≤ 2 cm (**F**), 2 cm < tumor size ≤ 5 cm (**G**), SCNEC (**H**), LCNEC (**I**)

After 1:1 PSM, a similar 5-year cumulative incidence of CSD was observed between the two cohorts (35.4% vs. 31.4%, *p* = 0.731). And there was no significant difference of cumulative incidence of OCD was found (9.7% vs. 19.7%, *p* = 0.133) (Fig. 3, Table 2). The subgroup analyses were repeated, and no significant link between chemotherapy and CSD was found in all the subgroups except the AJCC stage I cohort (all *p* > 0.05). In the multivariate competing risks regression analysis, there was no significant relationship between chemotherapy and CSD (HR, 0.79; 95% CI, 0.48–1.31; *p* = 0.36) (Table S1).

### Univariate and multivariate analysis

We then performed a second independent analysis. To build a prognostic model, we randomly assigned patients to two groups: training (70%, *n* = 297) and validation (30%, *n* = 127). The 1-, 3-, and 5-year CIF values of CSD in the training cohort were calculated using univariate analysis. Age, gender, grade, T stage, N stage, and tumor size were substantially associated with CSD. The significant variables (*p* < 0.1) were then discovered using the Fine-Gray proportional subdistribution hazards model's multivariate assessment. Age, gender, grade, T stage, N stage, and tumor size were found to be independent predictors of CSD in stage I–II GNEC patients following surgery in the multivariate competing risk analysis (Table 3).

**Table 3:** The cumulative incidences and multivariate subdistribution proportional hazards analysis on CSD

Characteristics	Cancer-specific death (%)				Subdistribution proportion hazards model		
	1-year CIF	3-year CIF	5-year CIF	*P* value	HR	95%CI	*P* value
Age				< 0.001			
≤ 60	0.01	0.1	0.13		Reference		
> 60	0.07	0.22	0.26		2.88	1.76–4.35	0.001
Gender				0.03			
Female	0.06	0.17	0.21		Reference		
Male	0.09	0.18	0.27		2.23	1.45–4.23	< 0.001
Race				0.354			
Non-White	0.05	0.13	0.2				
White	0.08	0.21	0.23				
Marital status				0.13			
Married	0.06	0.17	0.22				
Unmarried	0.11	0.24	0.28				
Grade				< 0.001			
II	0.05	0.15	0.2		Reference		
III/IV	0.13	0.26	0.27		2.12	1.55–3.23	< 0.001
Pathology				0.214			
SCNEC	0.04	0.12	0.14				
LCNEC	0.06	0.14	0.15				
T stage				< 0.001			
T1	0.05	0.13	0.17		Reference		
T2	0.06	0.12	0.15		1.03	0.55–1.88	0.66
T3	0.1	0.28	0.36		2.01	1.33–3.56	0.03
T4	0.26	0.5	0.63		7.8	3.55–16.13	< 0.001
N stage				< 0.001			
N0	0.04	0.13	0.19		Reference		
N1	0.08	0.3	0.32		2.55	1.64–3.88	< 0.001
N2/N3	0.08	0.32	0.38		3.05	1.46–7.32	< 0.001
Primary site				0.644			
Cardia	0.04	0.13	0.23				
Distal site	0.06	0.23	0.25				
Middle site	0.08	0.21	0.25				
Overlapping/NOS	0.05	0.14	0.21				
Tumor size				< 0.001			
≤ 2 cm	0.05	0.08	0.12		Reference		
2–5 cm	0.06	0.17	0.23		1.88	0.94–3.05	0.08
> 5 cm	0.11	0.28	0.34		2.21	1.11–3.74	0.03
RNE				0.582			
> 16	0.04	0.17	0.22				
0	0.19	0.24	0.27				
1–15	0.08	0.2	0.25				
Chemotherapy				0.09			
None	0.05	0.09	0.13				
Chemo	0.08	0.2	0.25				

*HR* hazard ratio

### Constructing and verifying the nomogram

**Fig. 4:**
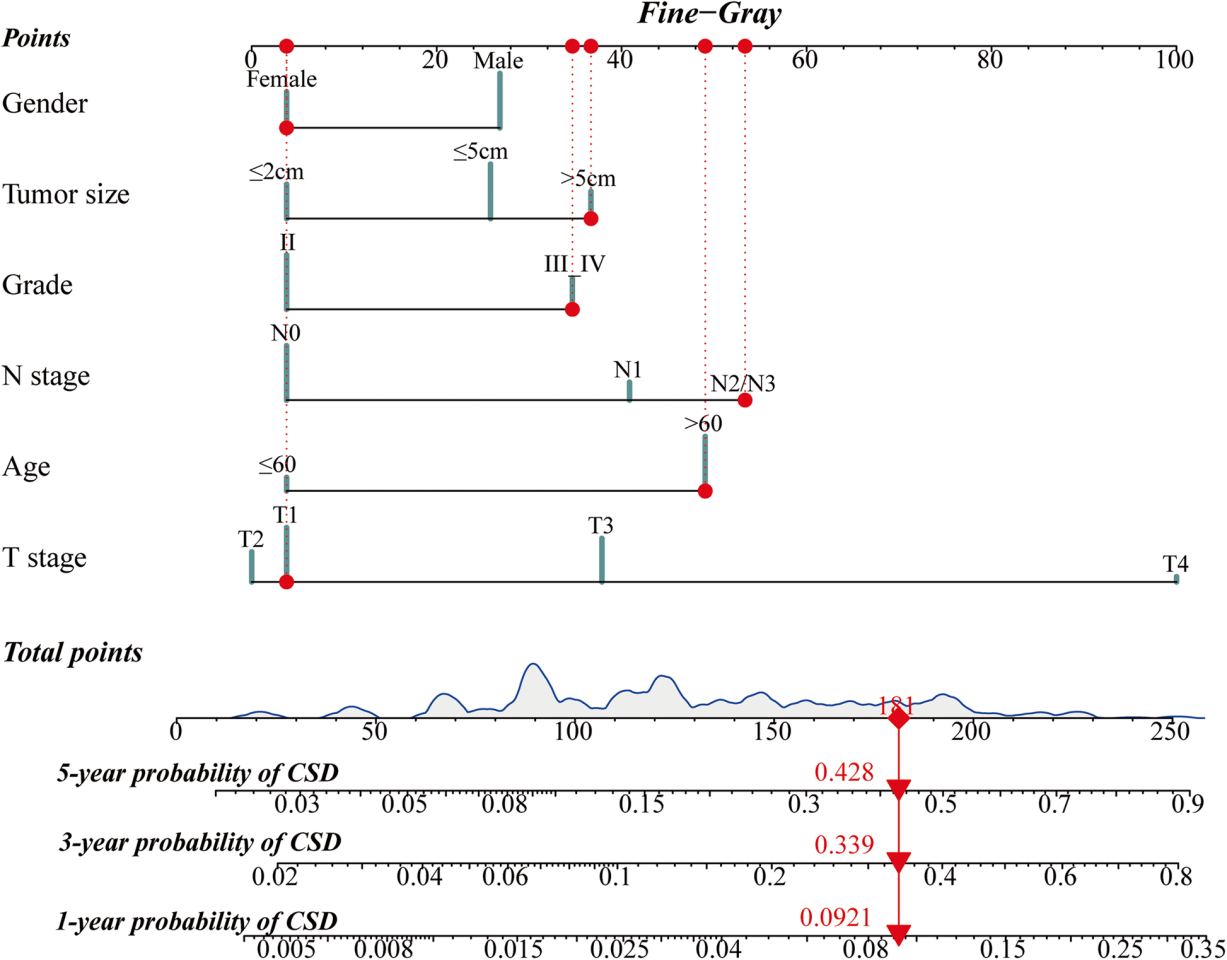
Nomogram based on the competing risk analysis to predict CSD probabilities at 1-, 3-, and 5-year in stage I–II GNEC patients

A competing event nomogram was then built based on the variables from the multivariate analysis to calculate the odds of CSD in 1-, 3-, and 5-year (Fig. 4). The predictive model included age, gender, grade, T stage, N stage, and tumor size. The total points were calculated by adding the scores for each patient’s prognostic characteristics, which clinicians could use to evaluate the likelihood of CSD at different time points for specific patients.

**Fig. 5:**
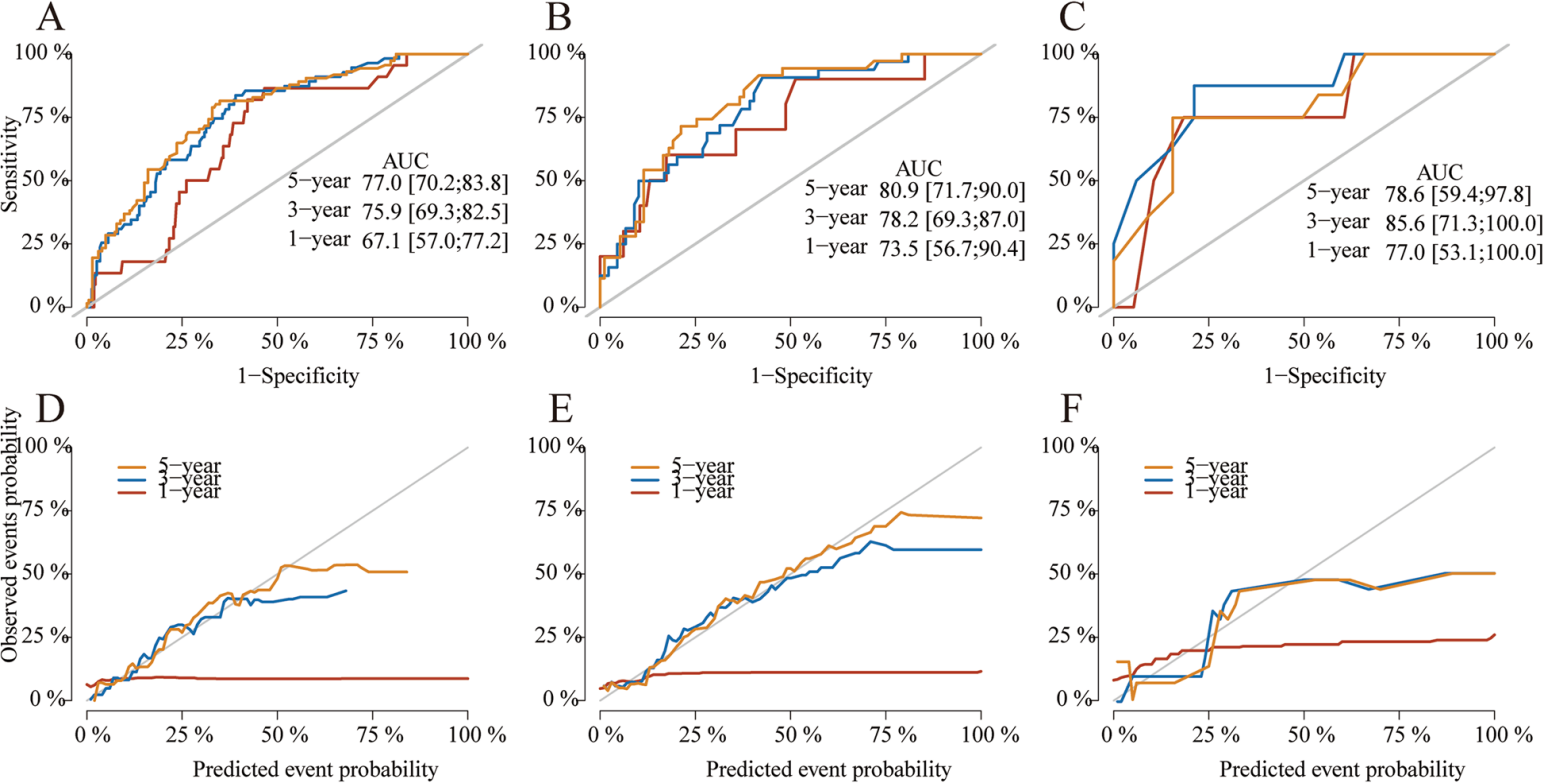
ROC curves at the 1-, 3-, and 5-year points in the training (**A**), internal validation (**B**), and external validation cohort (**C**). Calibration curves at the 1-, 3-, and 5-year points in the training (**D**), internal validation (**E**), and external validation cohort (**F**)

The model was tested with the internal and external validation cohort. The 1-, 3-, and 5-year AUC values were 0.770, 0.759, and 0.671 in the training cohort, 0.809, 0.782, and 0.735 in the internal validation cohort, 0.786, 0.856, and 0.770 in the external validation cohort, indicating high discrimination ability (Fig. 5A–C). Calibration plots were also used to test the model’s prediction accuracy, and we discovered that the expected and actual probabilities of CSD in the three datasets were relatively consistent (Fig. 5D–F). The above findings revealed our nomogram’s high credibility and good predictive potential.

## Discussion

In the research, we identified 424 GNEC patients from the SEER database and performed KM survival analysis and competing risk analysis. The results indicated that adjuvant chemotherapy could improve OS, but it could not improve CSS in these early-stage patients. When evaluating the actual effect of chemotherapy, cancer-specific survival was reported as a more reliable outcome than OS because OS can be diluted by OCD [16]. Furthermore, the presence of OCD might impede the detection of CSD, particularly in stage I–II patients who had a longer lifetime than advanced stage patients [17]. To investigate the potential competing risk of death, we further conducted competing risk analyses. After 1:1 PSM, a similar 5-year cumulative incidence of CSD was observed between the two cohorts (35.4% vs. 31.4%, *p* = 0.731). And there was no significant difference of cumulative incidence of OCD was found (9.7% vs. 19.7%, *p* = 0.133). Then the subgroup analyses and multivariate competing risks regression analysis confirmed no significant relationship between chemotherapy and CSD. Patients in the chemotherapy cohort presented higher proportion of male, grade III/IV, primary site of cardia, and tumor size over 5 cm, which had been confirmed as high-risk factors in previous studies [18]. Therefore, a lower percentage of high-risk variables in the no-chemotherapy cohorts and other probable comorbidities not recorded in the SEER database might help partially explain the difference in OCD between the two groups. Relatively long survival time also increases the risk of OCD, such as other malignancies and cardiovascular diseases.

GNEC is characterized by poor biological behavior and resistance to traditional chemotherapy [19]. For GNEC patients without distant metastasis, primary tumor resection remains the first choice. The Japanese Classification of Gastric Carcinoma recommended sequential adjuvant chemotherapy after radical surgery [6]. Several research supported the view of aggressive adjuvant chemotherapy. Xie et al. retrospectively analyzed clinical data from a single center, indicating that stage I-III GNEC patients could benefit from adjuvant chemotherapy (median survival time 43 months vs. 13 months, *p* = 0.026) [7]. Liu et al. investigated the prognosis of 43 GNEC patients, concluding that postoperative medical therapy was required. The median survival time was 44 months in the chemotherapy group and 15 months in the no-chemotherapy group. Besides, the research recommended the chemotherapy regimen of cisplatin plus Pacditaxel [20]. However, other research presented opposing views. A Chinese study recruited 804 GNEC patients from 21 centers between 2004 and 2016, indicating that stage I-II patients could not obtain improved prognosis after adjuvant chemotherapy based on platinum or 5-fluorouracil [8]. Therefore, there is still controversy about whether early-stage GNEC patients could benefit from adjuvant chemotherapy after radical surgery. Compared with the advanced stage, stage I–II GNEC patients tended to obtain more prolonged survival. However, no research has been conducted into the role of chemotherapy in these specific patients. Based on the competing risk analysis, this is the first study to look at the effect of chemotherapy in stage I–II GNEC patients and find that these patients cannot benefit from adjuvant chemotherapy.

Based on the results of competing risk analysis, a nomogram was proposed to individually predict the survival of stage I–II GNEC patients. Age, gender, grade, T stage, N stage, and tumor size were incorporated into the predictive model. N stage and T stage were considered high-risk factors by examining the max points of the integrated parameters. Previous research showed how these risk variables and GNEC were related. Hu et al. indicated a gradually rising trend of GNEC incidence in the past 40 years and constructed predictive nomograms. Age, grade, TNM stage, and primary tumor resection were significantly correlated with 3-, 5-, and 10-year OS [21]. Xu et al. conducted a comparative study between intestinal-type GC (IGC) and GNEC patients, demonstrating that GNEC patients presented a longer survival to ICG in the early-stage tumor. And age, gender, tumor size, AJCC stage, T stage, N stage, and surgery were considered the risk factors with the OS of GNEC patients [18]. As in our study, the multivariate competing risk analysis found older age, male gender, greater tumor diameter, and higher TNM stage as independent risk factors for CSD. Compared with the traditional multivariate regression model, the nomogram could visually present the individual probability of 1-, 3-, and 5-year CSD. The model was also verified in the validation cohort, illustrating the good predictive potential along with the high credibility of our nomogram.

## Limitation

There are several limitations in the research. To begin with, because this is a retrospective study, selection bias is unavoidable. Second, there is no “neuroendocrine cancer-specific” data in the SEER database. As a result, key critical characteristics for precise stagings, such as the Ki67 value and chemotherapy regimens, cannot be collected. Also, the external validation cohort is relatively small and further external multicenter prospective validations are required.

## Conclusion

By using competing risk analysis and 1:1 PSM analysis, we demonstrated that stage I–II GNEC patients could not benefit from adjuvant chemotherapy after surgery. De-escalation of chemotherapy should be considered for stage I–II GNEC patients. The nomogram could individually predict the 1-, 3-, and 5-year CSD in these patients and presented excellent prediction ability. Further research and RCTs are required to validate the conclusion.

## Supplementary Information

**Additional file 1: Figure S1.** The mean difference between the two cohorts.

**Additional file 2: Figure S2.** Cumulative incidence curves for the stage I-II GNEC patients in overall patients and subgroups before PSM. Overall patients (A), stage I (B), stage II (C), grade II (D), grade III/IV (E), tumor size≤2cm (F), 2cm<tumor size≤5cm (G), SCNEC (H), LCNEC (I).

**Additional file 3: Table S1.** The results of the multivariate subdistribution hazard model on CSD before and after PSM.

## Data Availability

The datasets generated and analyzed during this study can be found in the SEER database (https://seer.cancer.gov/), and any further questions can be directed to the corresponding author.
