# Supplementary figures and images for: De-escalating chemotherapy for stage I–II gastric neuroendocrine carcinoma? A real-world competing risk analysis
Source: World J Surg Oncol. 2023 May 6;21:142. doi: 10.1186/s12957-023-03029-2 (PMC10163728; doi:10.1186/s12957-023-03029-2)

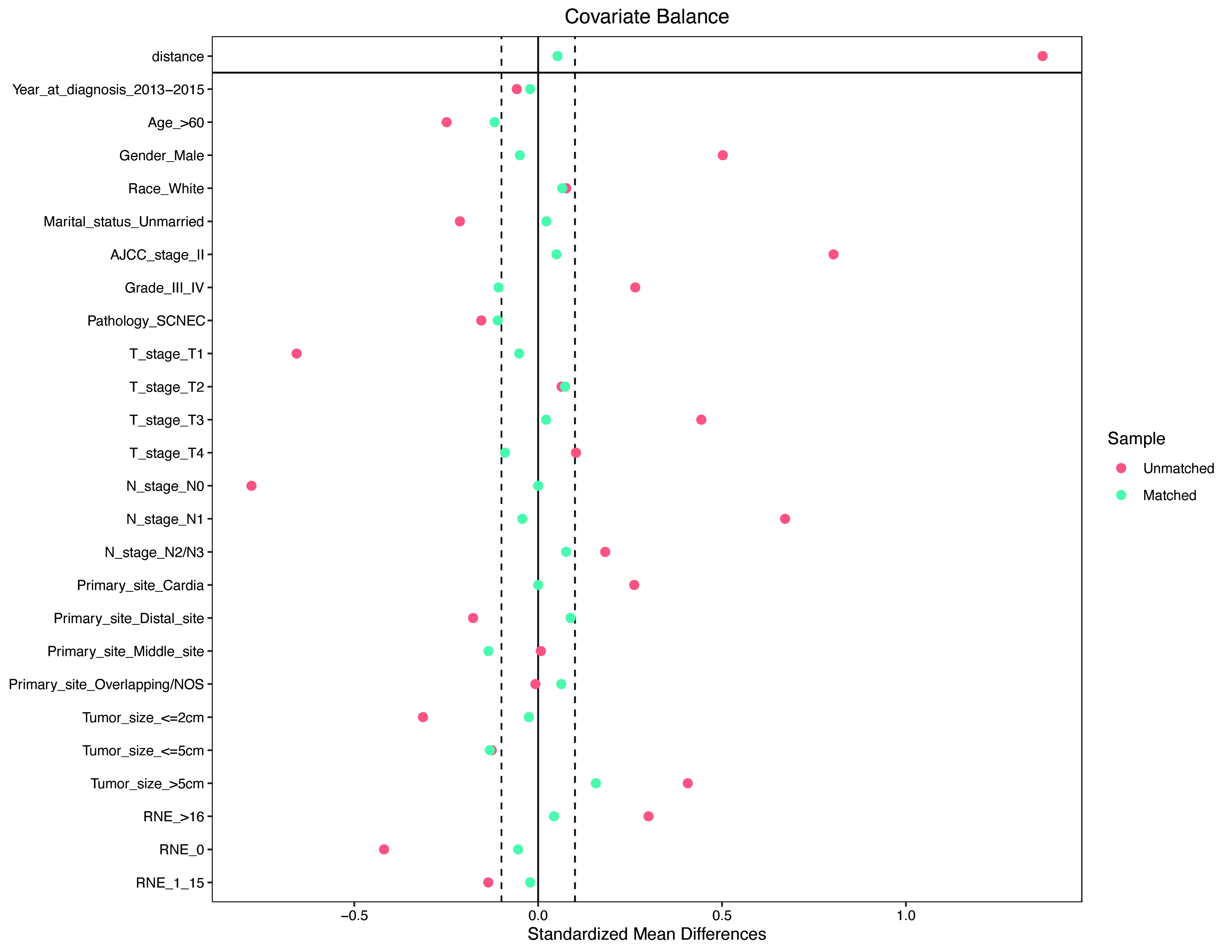

Supplement: Supplementary file 1 — Additional file 1: Figure S1. The mean difference between the two cohorts. [file 12957_2023_3029_MOESM1_ESM.tif]

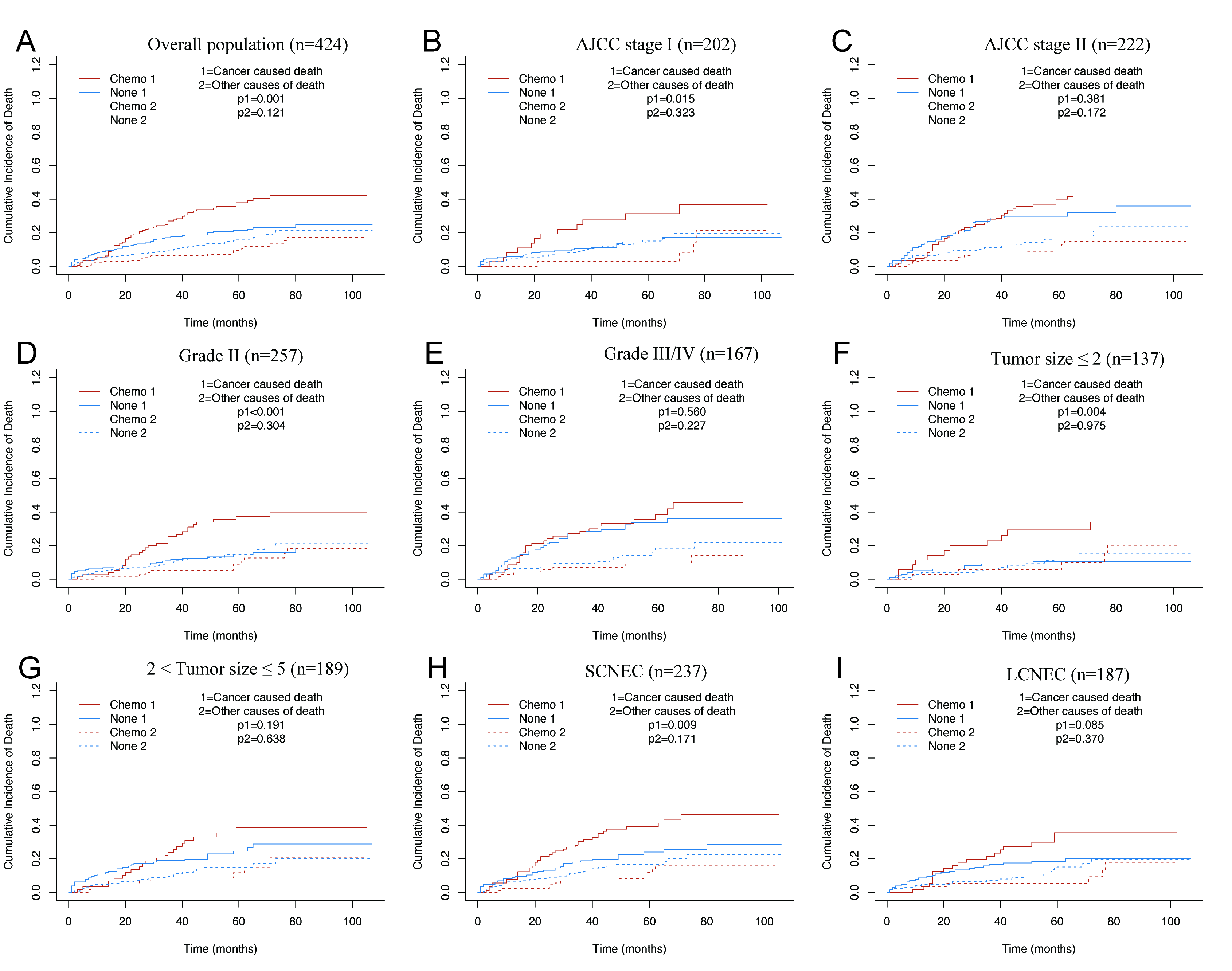

Supplement: Supplementary file 2 — Additional file 2: Figure S2. Cumulative incidence curves for the stage I-II GNEC patients in overall patients and subgroups before PSM. Overall patients (A), stage I (B), stage II (C), grade II (D), grade III/IV (E), tumor size≤2cm (F), 2cm<tumor size≤5cm (G), SCNEC (H), LCNEC (I). [file 12957_2023_3029_MOESM2_ESM.tif]
