# Supplementary material for: De-escalating chemotherapy for stage I–II gastric neuroendocrine carcinoma? A real-world competing risk analysis
Source: World J Surg Oncol. 2023 May 6;21:142. doi: 10.1186/s12957-023-03029-2 (PMC10163728; doi:10.1186/s12957-023-03029-2)
Supplement: Supplementary file 3 — Additional file 3: Table S1. The results of the multivariate subdistribution hazard model on CSD before and after PSM. [file 12957_2023_3029_MOESM3_ESM.docx]

| Table S1. The results of the multivariate subdistribution hazard model on CSD before and after PSM | | | | | | |
| --- | --- | --- | --- | --- | --- | --- |
| Characteristics | Before PSM | | | After PSM | | |
|  | HR | 95%CI | P value | HR | 95%CI | P value |
| Age |  |  |  |  |  |  |
| ≤60 | Reference |  |  | Reference |  |  |
| >60 | 2.62 | 1.42-4.82 | 0.002 | 2.44 | 1.07-5.53 | 0.033 |
| Gender |  |  |  |  |  |  |
| Female | Reference |  |  | Reference |  |  |
| Male | 1.92 | 1.17-3.16 | 0.01 | 1.61 | 0.81-3.18 | 0.18 |
| Race |  |  |  |  |  |  |
| Non-white | Reference |  |  | Reference |  |  |
| White | 0.97 | 0.66-1.44 | 0.89 | 1.03 | 0.59-1.81 | 0.91 |
| Marital status |  |  |  |  |  |  |
| Married | Reference |  |  | Reference |  |  |
| Unmarried | 1.41 | 0.91-2.19 | 0.12 | 1.17 | 0.61-2.24 | 0.64 |
| Grade |  |  |  |  |  |  |
| II | Reference |  |  | Reference |  |  |
| III/IV | 1.5 | 1.01-2.22 | 0.046 | 1.29 | 0.75-2.22 | 0.35 |
| Pathology |  |  |  |  |  |  |
| SCNEC | Reference |  |  | Reference |  |  |
| LCNEC | 0.91 | 0.61-1.36 | 0.65 | 1.02 | 0.54-1.91 | 0.95 |
| T stage |  |  |  |  |  |  |
| T1 | Reference |  |  | Reference |  |  |
| T2 | 1.73 | 0.91-3.31 | 0.097 | 2.14 | 0.76-6.05 | 0.15 |
| T3 | 4.58 | 1.62-12.95 | 0.004 | 5.18 | 1.03-25.99 | 0.046 |
| T4 | 15.36 | 4.53-52.08 | <0.001 | 17.64 | 2.85-109.15 | 0.002 |
| N stage |  |  |  |  |  |  |
| N0 | Reference |  |  | Reference |  |  |
| N1 | 2.06 | 1.17-3.62 | 0.012 | 2.27 | 0.98-5.24 | 0.056 |
| N2/N3 | 7.63 | 2.4-24.24 | 0.001 | 6.39 | 1.1-37.26 | 0.039 |
| Primary site |  |  |  |  |  |  |
| Cardia | Reference |  |  | Reference |  |  |
| Distal site | 0.7 | 0.29-1.71 | 0.44 | 0.52 | 0.12-2.16 | 0.36 |
| Middle site | 0.94 | 0.37-2.38 | 0.9 | 0.65 | 0.16-2.67 | 0.55 |
| Overlapping/NOS | 0.31 | 0.1-0.93 | 0.037 | 0.19 | 0.04-0.98 | 0.048 |
| Tumor size |  |  |  |  |  |  |
| ≤2cm | Reference |  |  | Reference |  |  |
| 2-5cm | 1.41 | 0.81-2.47 | 0.23 | 1.15 | 0.53-2.5 | 0.72 |
| >5cm | 1.87 | 0.98-3.56 | 0.057 | 1.45 | 0.61-3.43 | 0.4 |
| RNE |  |  |  |  |  |  |
| >16 | Reference |  |  | Reference |  |  |
| 0 | 4.01 | 2.07-7.8 | <0.001 | 3.49 | 1.32-9.22 | 0.012 |
| 1-15 | 1.25 | 0.84-1.88 | 0.27 | 1.06 | 0.56-1.99 | 0.86 |
| Chemotherapy |  |  |  |  |  |  |
| Chemo | Reference |  |  | Reference |  |  |
| None | 0.79 | 0.5-1.24 | 0.3 | 0.79 | 0.48-1.31 | 0.36 |
| HR Hazard ratio |  |  |  |  |  |  |
